# Enhanced crosslimb transfer of force-field learning for dynamics that are identical in extrinsic and joint-based coordinates for both limbs

**DOI:** 10.1152/jn.00485.2015

**Published:** 2015-11-18

**Authors:** Timothy J. Carroll, Aymar de Rugy, Ian S. Howard, James N. Ingram, Daniel M. Wolpert

**Affiliations:** ^1^Centre for Sensorimotor Performance, School of Human Movement and Nutrition Sciences, The University of Queensland, Brisbane, Australia;; ^2^Institut de Neurosciences Cognitives et Intégratives d'Aquitaine, Centre National de la Recherche Scientifique Unité Mixte de Recherche 5287, Université de Bordeaux, France;; ^3^School of Computing and Mathematics, Plymouth University, Plymouth, United Kingdom; and; ^4^Computational and Biological Learning Laboratory, Department of Engineering, University of Cambridge, Cambridge, United Kingdom

**Keywords:** motor learning, interlimb transfer, sensorimotor adaptation, coordinate frame

## Abstract

Humans are able to adapt their motor commands to make accurate movements in novel sensorimotor environments, such as when wielding tools that alter limb dynamics. However, it is unclear to what extent sensorimotor representations, obtained through experience with one limb, are available to the opposite, untrained limb and in which form they are available. Here, we compared crosslimb transfer of force-field compensation after participants adapted to a velocity-dependent curl field, oriented either in the sagittal or the transverse plane. Due to the mirror symmetry of the limbs, the force field had identical effects for both limbs in joint and extrinsic coordinates in the sagittal plane but conflicting joint-based effects in the transverse plane. The degree of force-field compensation exhibited by the opposite arm in probe trials immediately after initial learning was significantly greater after sagittal (26 ± 5%) than transverse plane adaptation (9 ± 4%; *P* < 0.001), irrespective of whether participants learned initially with the left or the right arm or via abrupt or gradual exposure to the force field. Thus transfer was impaired when the orientation of imposed dynamics conflicted in intrinsic coordinates for the two limbs. The data reveal that neural representations of novel dynamics are only partially available to the opposite limb, since transfer is incomplete even when force-field perturbation is spatially compatible for the two limbs, according to both intrinsic and extrinsic coordinates.

humans are able to adapt their motor commands to make accurate movements in novel sensorimotor environments, such as when visual information is distorted or when first using tools that alter limb dynamics. Moreover, some types of motor skill acquired with one limb can be performed well by the opposite limb, despite a lack of direct experience with the task (Carroll et al. 2008; Cook 1933; Hicks et al. 1983; Lee et al. 2010; Parlow and Dewey 1991; Perez et al. 2007; van Mier and Petersen 2006). Information about when and how this interlimb transfer occurs can provide insight into the neural representation of motor learning and may yield practical benefits by illustrating how the effect might best be harnessed in rehabilitation, workplace training, or sport. The issue has been addressed by studying reaching behavior in novel dynamic environments. However, there is contradictory evidence regarding whether learned representations of new dynamics obtained through experience with one limb are available to the opposite, untrained limb and if so, in which form they are available. Most previous studies involving adaptation to novel dynamic environments found non-negligible but asymmetric transfer; adaptation with the right limb benefited the left limb but not vice versa (Criscimagna-Hemminger et al. 2003; Galea et al. 2007; Wang and Sainburg 2004). However, Burgess et al. (2007) reported weak but significant left-to-right transfer. Adaptation to viscous curl fields improved performance with the opposite limb only when the field was defined according to end-point velocity in extrinsic space and not when the field direction was oriented according to the corresponding joint configurations of each limb (Criscimagna-Hemminger et al. 2003). In contrast, Wang and Sainburg (2004) found significant transfer when a dynamic perturbation that depended on joint kinematics was introduced by adding an inertial load to the forearm, and Galea et al. (2007) found intrinsic transfer in response to (assistive or resistive) viscosity manipulations that induced no directional or hand end-point errors. Moreover, Malfait and Ostry (2004) reported that transfer of a viscous force field occurred only when the perturbation was introduced abruptly to induce large initial errors but not when there was a gradual introduction over multiple trials.

A key limitation common to all of these studies is that transfer was inferred on the basis of the size of the kinematic errors caused by dynamic perturbation. This prevents definitive information about the degree to which a new sensorimotor map of the novel dynamics obtained with one limb is available to its opposite, because multiple processes can contribute to a more direct end-point path to the target in the presence of a force field. For example, the kinematic errors caused by perturbing forces can be reduced by predictive compensation for the imposed dynamics, changes in limb impedance that resist the effects of physical perturbations, and/or changes in feedback gains that correct trajectory errors more effectively. To address these issues, Joiner et al. (2013) recently re-examined interlimb transfer of a force-field adaptation by measuring the lateral forces made by subjects against the walls of a virtual force channel, which constrained reaching movements to follow a straight path to the target. Because in these conditions, kinematics are constrained to enforce task achievement, adaptive limb impedance tuning and feedback corrections should contribute little to behavior, and the lateral forces measured should reflect the degree to which the feedforward motor plan is appropriate to counter the imposed dynamics. The authors found that exposure to an extrinsically defined viscous force field with the right arm resulted in small but significant transfer to the left arm (∼12%), irrespective of whether the field was introduced abruptly or gradually. This indicates that neural representations of newly encountered dynamics are stored in a network that is, at least partially, accessible to the opposite limb but leaves a number of questions unresolved.

In particular, the finding that transfer occurs according to extrinsic coordinates is perhaps surprising, given the historical view that novel dynamics are represented primarily according to a joint-based coordinate frame (Shadmehr and Mussa-Ivaldi 1994). However, recent data indicate that learned dynamics do not generalize globally, according to any single coordinate frame, and appear to be represented locally with respect to the specific spatial parameters encountered during learning and/or according to a combination of multiple spatial reference frames (Berniker et al. 2014; Parmar et al. 2015). Whereas these studies examined the coordinate system in which the brain learns and generalizes, there is also a long history of studies that has examined the coordinate systems in which natural movements are represented. These studies have tended to follow one of two approaches. In the first approach, the coordinate frames of movement representation are inferred from the analysis of errors and variability of movement [e.g., Flanders and Soechting (1995); Soechting and Flanders (1992); cf. McGuire and Sabes (2009)]. The second approach is neurophysiological, in which correlations between neural activation and movement parameters are used to infer the coordinate systems of neural representations [for review, see Buneo and Andersen (2006); Crawford et al. (2004); Sabes (2011)]. Both approaches provide evidence that motor control involves multiple or mixed intrinsic/extrinsic coordinate systems. Here, we ask a related but distinct question: to what extent does the coordinate system representation of a novel task determine the ability of subjects to transfer learning from one limb to the other? In particular, the potential for a mixed intrinsic/extrinsic internal representation of learned dynamics raises the possibility that transfer between limbs depends on the degree to which the perturbation is aligned, according to different coordinate systems for the two limbs.

Crucially, lateral perturbations have conflicting effects in joint-based and extrinsic coordinates for the left and right arm, due to their mirror symmetry (Fig. 1*C*). Therefore, only very simple one-dimensional (1D), horizontal force fields (in which forces must only act in the sagittal direction and only depend on the sagittal movement of the hand) can be the same in both joint and extrinsic coordinates for the two limbs. If adaptation is encoded simultaneously in multiple reference frames, then new sensorimotor representations developed to compensate for laterally oriented force fields may be incompatible with opposite-limb use, even if their neural substrate is accessible to both arms. The degree to which dynamic adaptations are bilaterally available may therefore have been underestimated in previous studies that involved frontal and transverse plane reaching. Indeed, we recently showed that learning in an isometric visuomotor rotation task transferred strongly between limbs when intrinsic and extrinsic reference frames were aligned for the two limbs but not when there were reference-frame misalignments (Carroll et al. 2014).

**Fig. 1.**
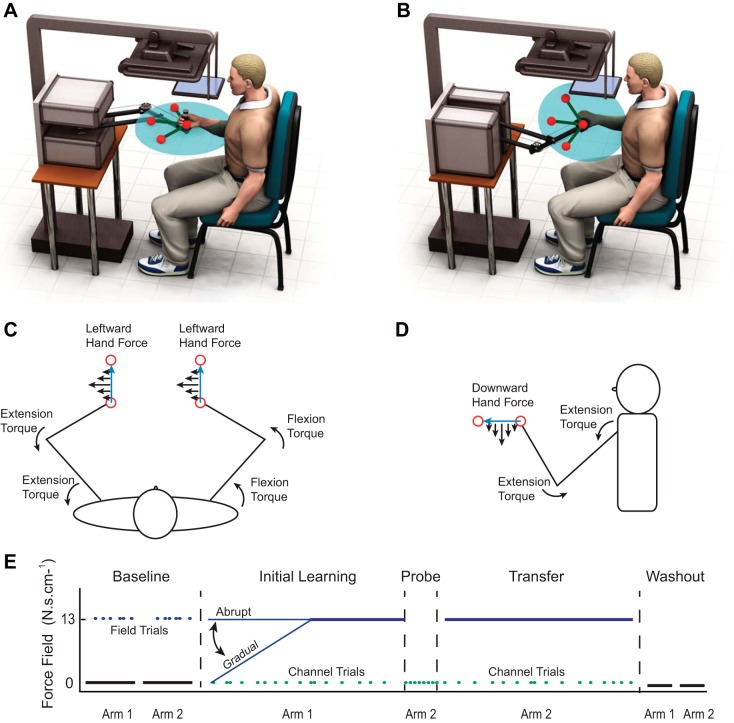
Schematic illustrations of the experimental setup. *A* and *B*: transverse and sagittal plane reaching conditions, respectively. *C* and *D*: the orientation of joint torques imposed upon the left and right arms by a given curl force field defined in extrinsic coordinates for the transverse and sagittal plane-reaching conditions, respectively. The joint torques are identical for both limbs during sagittal reaching but differ for transverse reaching. *E*: the time course of each experiment and the composition of the various experimental phases. Force-field trials, blue; null field trials, black; channel trials, green. Only 15 of the 60 channel trials completed are shown per phase (initial learning and transfer) for clarity.

Here, we examined crosslimb transfer of force-field adaptation during reaching in the midsagittal plane, where force fields always have the same representation in both joint and extrinsic coordinates. Specifically, we compared transfer of force-field adaptation for movements in the transverse (horizontal) and midsagittal planes. We found moderate transfer (∼25%) in both directions between left and right limbs for sagittal plane reaching, which was significantly greater than that observed for the horizontal plane task (≤10%). The data reveal that neural representations of novel dynamics are only partially available to the opposite limb, since transfer is incomplete even when force-field perturbation is spatially compatible for the two limbs according to both intrinsic and extrinsic coordinates.

## MATERIALS AND METHODS

### 

#### Participants.

Forty-eight right-handed (Oldfield 1971) people with no reported neurological conditions and with normal or corrected-to-normal vision participated in the experiment (16 women, 32 men; 19–54 yr old). The sample was of convenience, determined by the characteristics of those individuals who responded to advertisements inviting study participation. All participants gave informed consent before the experiment, which was approved by the local Ethics Committee and conformed to the Declaration of Helsinki.

Each person performed a series of reaching movements in a virtual-reality environment that provided continuous 3D feedback of hand and target position. All subjects adapted to a viscous curl field during reaching movements made in a plane (either the transverse or the sagittal plane for different participants). The generalization of learning to the contralateral arm was then examined in the same plane of movement for the same viscous curl field (extrinsically defined) as initial learning.

Six groups of subjects (Table 1) performed different conditions (*n* = 8/group) that differed in the arm used for initial learning (left or right), the plane used for the movement (sagittal or transverse), or the rate of introduction of the force field during initial learning (gradual or abrupt). Three groups made movements in the sagittal (vertical) plane (Fig. 1, *B* and *D*) and three in the transverse (horizontal) plane (Fig. 1, *A* and *C*). One transverse- and one sagittal-reaching group each adapted to a viscous curl field with the right arm when the field was introduced abruptly. Another transverse- and another sagittal-reaching group each adapted to a viscous curl field with the right arm when the field was introduced gradually. The third transverse- and sagittal-reaching groups each adapted with the left arm when the field was introduced abruptly. We chose not to examine gradual initial learning with the left arm, as the three other conditions within each plane allowed us to perform the key comparisons that relate to previous work. That is, we compared the following: *1*) transfer after abrupt learning from left to right and from right to left and *2*) transfer from right to left after both gradual and abrupt initial learning.

**Table 1.** Composition of experimental groups

Group	Initial Learning Arm	Transfer Arm	Force-Field Plane	Force-Field Introduction	Sample Size
LR abrupt Sag	Left	Right	Sagittal	Abrupt	8
RL abrupt Sag	Right	Left	Sagittal	Abrupt	8
RL gradual Sag	Right	Left	Sagittal	Gradual	8
LR abrupt Hor	Left	Right	Horizontal	Abrupt	8
RL abrupt Hor	Right	Left	Horizontal	Abrupt	8
RL gradual Hor	Right	Left	Horizontal	Gradual	8

LR, left to right; Sag, sagittal; RL, right to left; Hor, horizontal.

#### Reaching task.

Subjects made planar reaches, while grasping the handle of a robotic manipulandum (vBOT). The vBOT is a modular, 2D planar manipulandum comprising a two-link carbon fiber arm arrangement, which is driven by motors operating on timing pulleys [for full detail of the apparatus, see Howard et al. (2009)]. It was mounted on a turntable that allowed its planar operating space to be rotated between the vertical and horizontal planes. For vertical plane operation, the weight of the handle and robot arm was actively compensated for by the robot motors. At the center of the workspace, the effective end-point mass in the sagittal condition was 500 g, and therefore, the weight compensation required ∼5 N. As the motors are capable of generating 40 N at the end point, this compensation, together with force field, never saturated the motor. In different phases of the experiment, the robot was used to generate one of three dynamic environments: *1*) a null field, in which the robot imposed no additional forces other than weight compensation in the vertical-reaching conditions, *2*) a viscous curl field, and *3*) a force channel that constrained reaches to follow a straight path to the target. For the viscous force field, the force generated by the vBOT was given by

$$\left[\begin{array}{c} F_{x}\\ F_{y} \end{array}\right]=k\left[\begin{array}{cc} 0 & -1\\ 1 & 0 \end{array}\right]\left[\begin{array}{c} \dot{x}\\ \dot{y} \end{array}\right]$$

where *k* was set equal to ±13 N·m^−1^·s. The sign of *k* determined the direction of the force field (clockwise or counterclockwise), which was counterbalanced across subjects. Channel trials were used to assess feedforward adaptation. In a channel trial, the movement was confined to a simulated mechanical channel on a straight path to the target with a spring constant of 4,000 N/m orthogonal to the wall (Howard et al. 2009).

There was one home target and three radial targets, arranged 14 cm away. The angle between neighboring radial targets and the home target was 60°. Each reach to a target was followed on the next trial by a reach back to the home target so that the six movement directions were distributed symmetrically throughout 360° (see Fig. 1, *A* and *C*). Subjects were seated such that the home target was ∼20 cm in front of the body and aligned with the center of the sternum. Force channel trials were only scheduled for reaches between the origin and the central radial target, both out and back. For channel trials in both sagittal and transverse plane conditions, therefore, the robot constrained the hand to follow the same horizontal path toward or away from the body in the midsagittal plane.

Participants were instructed to make a quick movement as soon as each target appeared and to stop sharply on the target. A tone signaled target presentation. Targets were extinguished when acquired, determined as the time when the cursor was within the target circle continuously for 100 ms. If targets were not acquired within 2 s, then a low-pitched warning tone was sounded, an error message was displayed on the screen, and the trial was repeated. The next target appeared 2–4 s later. The warning tone and text feedback (“too fast” or “too slow”) were presented after any trial in which the peak velocity fell outside of the desired range of 65–75 cm/s, but these trials were not repeated.

Force and position data were sampled at 1 kHz. Visual feedback was provided using a 21-in. cathode ray tube computer monitor (Dell UltraScan P1110), running at 100 Hz, mounted above the vBOT, and projected to the subject via a mirror. The mirror prevented vision of the manipulandum and the subject's arm. Stereoscopic images were provided to each eye at 50 Hz via liquid crystal display shutter goggles (CrystalEyes; StereoGraphics, San Rafael, CA) to provide appropriate depth cues in the 3D workspace. Targets and home positions were displayed as regular 20-sided wire polygons of radius 1.2 cm, with a solid sphere of radius 0.3 cm displayed at the center. The hand position was represented as a 0.5-cm radius red sphere. Because the monitor size prevented veridical display of the full workspace with 14 cm target distances, the virtual visual feedback was linearly reduced in scale by six/seven (i.e., 14 cm targets appeared 12 cm away). The scaling was imperceptible to the experimenters during pilot testing and to participants.

#### Experimental design.

After a brief familiarization with the baseline-reaching task with both arms, subjects completed the experimental protocol summarized in Fig. 1*E*. They first performed 96 reaches with each arm in the “baseline” phase of the experiment, starting with the arm that they would use for initial learning. Trials were performed in 16 blocks, with each block comprising one reach to every target (i.e., 6 reaches), with the order of out-and-back movements to each target pair randomized. Ninety of these trials were completed in a null field. The curl field was applied for the remaining six trials—one trial to each target in six randomly selected blocks, excluding the first two blocks. These random field trials were included to probe the kinematic consequences of the force field before any learning.

The baseline was followed by 420 “initial learning”-phase trials with the hand first used in baseline. The initial learning trials were performed in 30 blocks, each consisting of 14 trials, 2 trials to each target in the force field, and 2 trials between the origin and the central radial target (i.e., out and back) in the force channel (randomly ordered). For subjects assigned to each of the four “abrupt” initial learning conditions, the maximum curl-field strength was applied throughout all blocks. For those in the two “gradual” initial learning conditions, the field strength was linearly increased from zero in the first block to maximal field strength by the 15th block and held constant for the remaining 15 blocks. Immediately after adaptation with one hand, all subject groups performed eight reaches with the contralateral arm between the origin and the central radial target (4 out and 4 back) in the force channel, followed by 420 “transfer” trials at full field strength, according to the same pseudorandom target schedule as in the initial learning phase. The first eight channel trials were included to probe the degree to which adaptation with one arm resulted in feedforward compensation for the learned dynamics in the contralateral arm before systematic exposure to the force field. Finally, subjects performed 48 trials in a null field with the arm used in the initial adaptation, followed by 48 trials with the transfer arm to probe the kinematic aftereffects of adaptation. Short rest breaks were scheduled whenever subjects switched arms and three times during the initial adaptation- and transfer-phase blocks.

#### Data analysis.

A fifth-order Butterworth filter, with a low-pass cutoff frequency of 6 Hz, was applied to position, force, and velocity data before analysis. The reaction time for movement initiation after target presentation was measured as the period before the time when speed first exceeded 2 cm/s. Movement time was calculated between movement initiation and the time when hand speed first dropped below 5 cm/s after peak speed. For each trial, we calculated the peak speed of the movement, the maximum perpendicular distance by which the hand deviated from a straight path from the home to the center of the target in the direction of the imposed force, and the signed perpendicular error at time of peak hand speed. For channel trials, we calculated the mean perpendicular force exerted against the channel wall in the period corresponding to time of peak hand speed ± 70 ms, divided by the field constant, times the average hand speed, over the same time window. We multiplied this measure by 100 so that it corresponded to the percentage of full adaptation to the force field (negative values correspond to forces that were in the same direction as the force field) and term this measure “percentage field compensation.”

We also calculated two additional measures of channel-trial behavior to check that the findings were robust across different summary methods. First, we measured the average lateral force exerted over the entire movement duration of each channel trial, both in absolute units and relative to average hand speed. Next, we measured the slope of linear regression (with intercept forced through 0) for the ideal force trajectory (speed × force-field constant) vs. the applied force [as per Smith et al. (2006)]. The data were essentially identical for the three methods, so we focus on lateral forces over the 140-ms window throughout the manuscript. Percentage transfer was calculated as the difference between the percentage field compensation for the eight probe trials performed with the untrained contralateral arm immediately after initial adaptation divided by percentage field compensation in the last five blocks of the initial learning phase. The ezANOVA function in R was used to perform mixed repeated-measures ANOVAs (type II), with arm as the within-subjects factor (i.e., initial learning arm vs. transfer arm) and reaching plane orientation (i.e., sagittal vs. transverse) and exposure schedule (i.e., left to right abrupt vs. right to left abrupt vs. right to left gradual) as between-subjects factors. Pair-wise *t*-tests with Holm corrections for multiple comparisons were used to assess effects of a priori interest. Data are summarized as means with 95% confidence intervals in both text and figures. Holm corrected probabilities are cited in the text, and the significance level was set as α < 0.05. Note that exact *P* values are reported where possible, after multiplication by the relevant Holm correction factors (output from the p.adjust function in R). Exceptions include cases where *P* < 0.001 and where more than one contrast is being summarized (in which case, *P* is reported as less than the highest *P* value among the relevant contrast set to 2 decimal places). Whenever significant ANOVA effects are cited, all higher-order interactions are explicitly reported or nonsignificant.

## RESULTS

### 

#### Reaching characteristics.

The basic characteristics of the reaching movements are summarized in Table 2. Participants effectively matched the target hand speed of 70 cm/s in all conditions. Speeds were consistent throughout the experiment, except for a slight tendency for faster movements in the first block of the transfer phase rather than the initial learning phase (mean adaptation peak speed, 67.9 ± 1.8 cm/s; mean transfer peak speed, 71.4 ± 1.9 cm/s; F_1,42_ = 25.7, *P* < 0.001), which was significantly stronger for the transverse than the sagittal plane conditions (plane of movement by time interaction; F_1,42_ = 5.0, *P* = 0.03). Movement times were significantly longer during the sagittal rather than transverse plane reaching (plane of movement main effect; F_1,42_ = 14.3, *P* < 0.01), which may relate to additional time required for error corrections in the sagittal plane, since kinematic variability was greater in this condition (see below). Average reaction times from target presentation to movement onset were short and similar between conditions.

**Table 2.** Basic reaching-task characteristics

Group	Peak Speed, cm/s	Movement Time, ms	Reaction Time, ms
LR abrupt Sag	70 ± 4	520 ± 32	229 ± 17
RL abrupt Sag	70 ± 2	505 ± 16	221 ± 20
RL gradual Sag	70 ± 3	509 ± 25	236 ± 16
LR abrupt Hor	71 ± 2	479 ± 21	218 ± 16
RL abrupt Hor	70 ± 3	489 ± 19	230 ± 41
RL gradual Hor	71 ± 2	473 ± 20	226 ± 20

Group averages and 95% confidence intervals for each parameter averaged across all trials in the experiment per subject.

#### Force channel results.

**Fig. 2.**
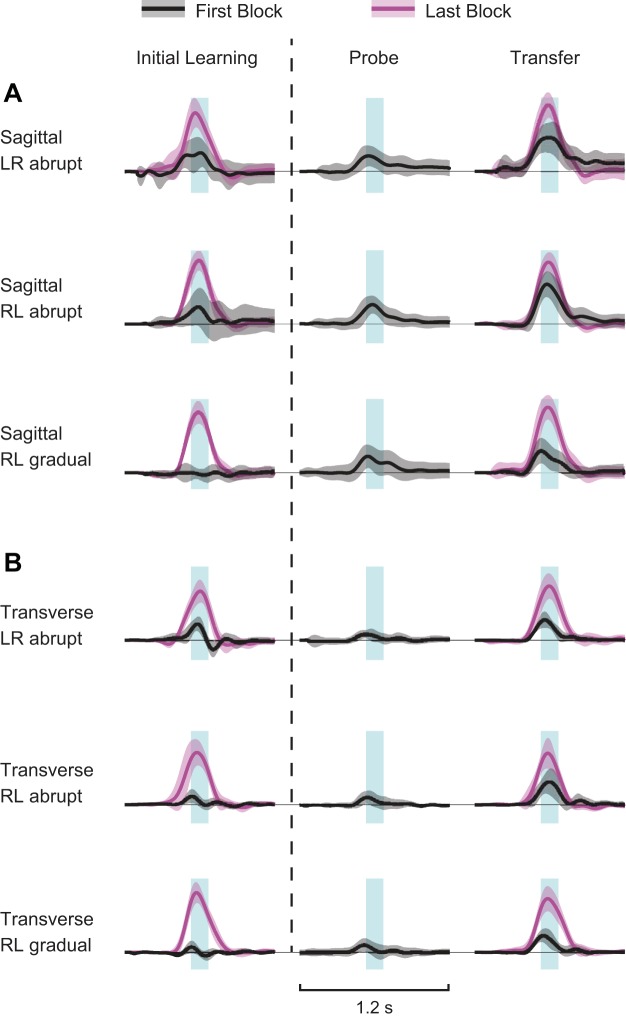
Average time-series plots of channel forces divided by peak hand speed for each trial for sagittal (*A*) and transverse (*B*) groups. Error bars show 95% confidence intervals. Trials were aligned according to the time of peak hand speed and averaged from 600 ms before to 600 ms after the time of peak speed. The blue boxes show both the time window (width) around peak hand speed used to measure force-field compensation (±70 ms) and the force magnitude (height) required to compensate perfectly the imposed field (i.e., 13 N·m^−1^·s) at peak hand speed (i.e., ∼0.7 m/s). Thus the unit of the ordinate of each plot is the percentage of the force required to compensate the imposed field at peak hand speed. Scale is specified by the height of the blue box as 100%. The first and last block of initial learning and transfer phases is shown, as well as the probe trials conducted just before the transfer phase for the “untrained” hand. LR, left to right; RL, right to left.

Figure 2 shows average time-series plots for the peak speed-normalized lateral forces exerted against the channel wall for each group in the first and last blocks of initial learning and transfer phases of the experiment and for the probe trials performed before force-field exposure with the arm used in transfer. The magnitude of the positive forces exerted increased during the adaptation phase of the experiment for all groups, indicating that subjects learned to produce feedforward motor commands appropriate to partially compensate for the imposed dynamics.

**Fig. 3.**
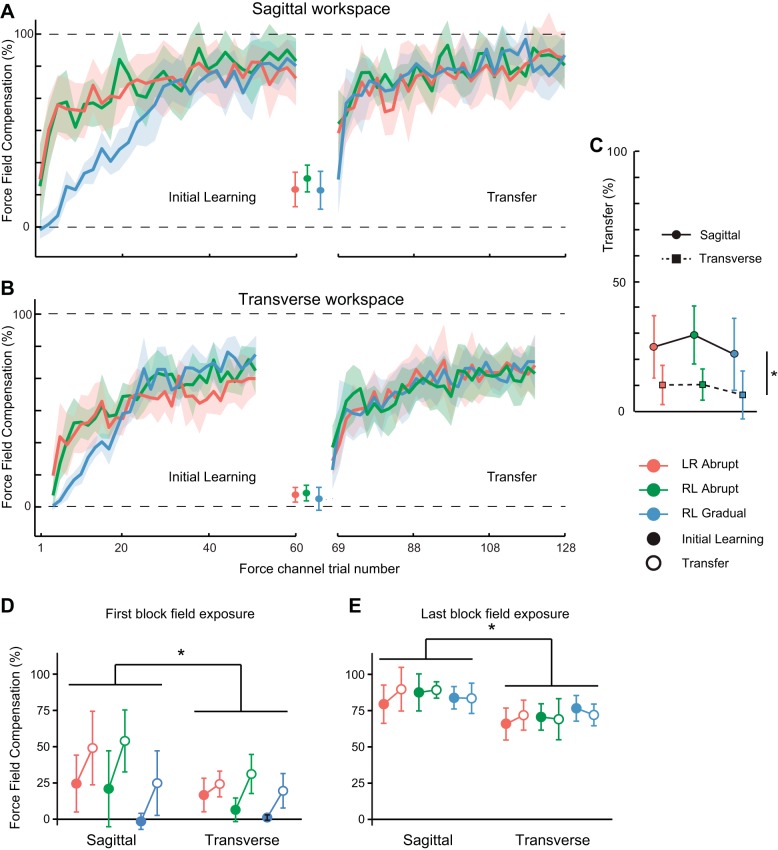
Force-field compensation. Averages and 95% confidence intervals for average percentage force-field compensation at peak hand speed ±70 ms in each condition for sagittal (*A*) and transverse (*B*) plane reaching. The 3 points plotted between initial learning- and transfer-phase data show average probe-trial forces. *C*: average and 95% confidence intervals for percentage transfer after opposite-limb adaptation for all 6 conditions. Lines join initial learning- and transfer-phase data for each condition. *D*: averages and 95% confidence intervals for percentage force-field compensation produced in the first initial learning and transfer blocks for all 6 conditions. *E*: averages and 95% confidence intervals for percentage force-field compensation produced in the last initial learning and transfer blocks for all 6 conditions. *P < 0.05, statistically significant main effects for transfer or force-field compensation between sagittal and transverse planes of movement.

Figure 3, *A* and *B*, shows the percentage force-field compensation for the channel trials over the course of the experiments for the six groups. In the first initial learning block, when the field was introduced abruptly, there was no difference in force compensation between sagittal and transverse groups (t_30_ = 1.5, *P* = 0.14; Fig. 3*D*), but the compensation became significantly greater for the sagittal rather than the transverse plane groups when averaged across the first five initial learning blocks (t_30_ = 3.6, *P* = 0.001). At the end of initial learning, the percentage compensation was greater for the sagittal plane groups (84 ± 6%) compared with the transverse plane groups (71 ± 5%; F_1,42_ = 22.5, *P* < 0.001; Fig. 3*E*). These data indicate that the adaptation with the arm first exposed to the force field was both more rapid and more complete for sagittal plane than transverse plane reaching.

The degree of force-field compensation exhibited by the contralateral arm in channel trials performed immediately after initial learning is the critical measure to assess interlimb transfer of newly acquired dynamics. The percentage transfer was significantly greater for sagittal (26 ± 6%) rather than transverse plane groups (9 ± 4%; F_1,42_ = 21.4, *P* < 0.001; Fig. 3*C*) and was significantly greater than zero for sagittal plane reaching, irrespective of which limb originally adapted to the field and whether the perturbation was introduced gradually or abruptly (all t_7_ > 3.7, all *P* < 0.02). Percentage transfer was significantly greater than zero for both transverse plane groups that initially adapted with the field introduced abruptly (both t_7_ = 3.2, *P* < 0.03) but not when the field was introduced gradually (t_7_ = 1.6, *P* = 0.15). Similar results were obtained for channel forces averaged over the entire movement duration, except that the nominal difference from zero for the gradual transverse plane group was also statistically significant (all t_7_ > 2.5, all *P* < 0.05). It is unlikely that factors other than transfer of adaptation from the initial limb contributed substantially to these nonzero force channel results. Any inherent biases to apply forces in one direction or the other when reaching to the channel targets should sum to zero, because we counterbalanced the curl-field directions. Indeed, for the first block of adaptation in the gradual conditions, when the imposed force field was negligible, the mean forces were close to zero for both sagittal and transverse plane groups (both t_7_ < 1.3, both *P* > 0.79; Fig. 3*D*). The result is also not due to the specific analysis method, as channel forces averaged over the entire movement duration were also negligible for the first-block gradual conditions (sagittal, 1.3 ± 7.5%; transverse, −0.6 ± 2.8%). This also argues against a possible contribution to probe-trial performance from the six field trials randomly presented during the baseline-reaching blocks.

It is also of interest to assess whether the magnitude of lateral forces applied in channel trials within the first initial learning block differed from that in the first transfer-phase block. This comparison does not provide a pure measure of transfer, because there was opportunity for sensorimotor adaptation to correct errors experienced in any force-field trials scheduled before the channel trials in these first blocks. The results could therefore reflect a combination of learned dynamic compensation, obtained purely through contralateral limb adaptation (i.e., transfer), and any effect of contralateral limb adaptation on the initial rate at which force-field compensation developed. There was a general tendency for larger channel forces in the first transfer-phase block than the first initial learning-phase block when pooled across all conditions (initial learning vs. transfer main effect; F_1,42_ = 39.5, *P* < 0.001; Fig. 3*D*), but none of the initial learning vs. transfer pair-wise contrasts was significant for any individual condition when corrected for multiple comparisons (all t_7_ < 3.7, all *P* > 0.07). Channel forces were also larger after contralateral limb adaptation when averaged over the first five transfer-phase blocks (time main effect; F_1,42_ = 117.1, *P* < 0.001). Although there was also a significant time-by-condition interaction effect (F_1,42_ = 19.4, *P* < 0.001) that reflected dramatic increases in channel forces for the gradual conditions, the initial learning vs. transfer pair-wise contrasts were significant for all individual conditions (all t_7_ > 2.9, all *P* < 0.05) except for the group that initially adapted with the left hand to an abrupt field in the transverse plane (t_7_ > 1.2, *P* = 0.27). Taken together, the data show that learned adaptation to novel dynamics transfers, at least partially (10–25%), to the contralateral arm.

#### Kinematic error results.

**Fig. 4.**
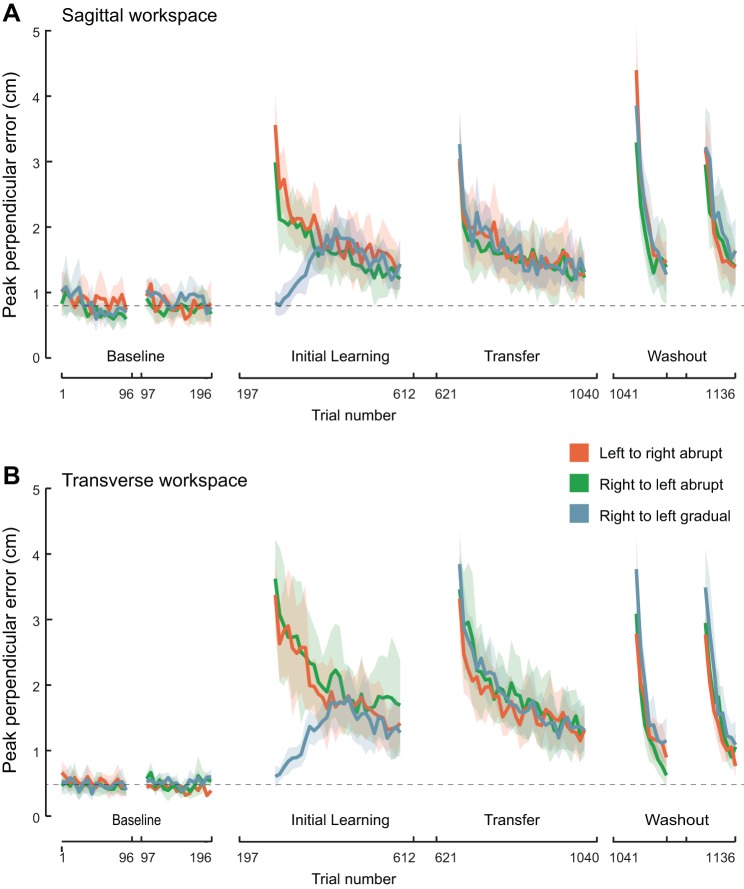
Kinematic analysis. Averages and 95% confidence intervals for peak perpendicular errors from a straight line path from the origin to the target for sagittal (*A*) and transverse (*B*) conditions.

Baseline reaching in the null field was significantly less accurate during sagittal plane than transverse plane reaching (last-block sagittal peak perpendicular error, 0.71 ± 0.08 cm; transverse peak perpendicular error, 0.45 ± 0.05 cm; F_1,42_ = 31.7, *P* < 0.001; Fig. 4, *A* and *B*). The trial-to-trial variability of the peak perpendicular error was also greater for sagittal than transverse conditions in baseline trials (SD of sagittal perpendicular errors, 1.56 ± 0.10 cm; SD of transverse peak perpendicular errors, 0.91 ± 0.05 cm; F_1,42_ = 99.2, *P* < 0.001). In contrast, perpendicular errors induced by the force field in the six field trials, randomly interspersed within the baseline blocks, were significantly larger for the transverse plane groups (sagittal error, 4.4 ± 0.2 cm; transverse error, 4.8 ± 0.2 cm; F_1,42_ = 5.9, *P* = 0.02). There was also a significant plane of movement by time interaction (F_1,42_ = 7.7, *P* = 0.01), reflecting nonsignificant trends for lower errors with the transfer arm rather than the initial learning arm during baseline field trials (initial learning error, 4.4 ± 0.3 cm; transfer error, 4.3 ± 0.3 cm; t_143_ < 0.7, *P* = 1.0) and for greater errors with the transfer arm for the transverse groups (initial learning error, 4.6 ± 0.3 cm; transfer error, 4.9 ± 0.3 cm; t_143_ = −1.6, *P* = 1.0). As there was no tendency for improved performance as a consequence of these force-field probes (overall mean error on first field probe trial, 4.52 ± 0.35 cm; sixth field trial, 4.54 ± 0.38 cm; F_5,210_ = 0.7, *P* = 0.64; no significant first- vs. last-trial differences for any group; all t_7_ < 1.6, all *P* = 1.0, despite a significant 3-way plane of movement by trial-by-condition interaction effect, F_10,210_ = 2.2, *P* = 0.02), the baseline data suggest that before learning, limb impedance or rapid-feedback gains were more effective for compensating the effects of vertical rather than horizontal dynamic perturbations. The differences in performance between tasks could be due to the intrinsic biomechanics of the limb or to differences in feedforward or feedback muscle activity. In this regard, the fact that subjects were required to support the entire weight of their limb actively in sagittal plane reaching, but could partially support their arm weight by grasping the horizontally constrained robot handle in transverse plane reaching, may be relevant.

Consistent with the conclusion that the field trials randomly applied during the baseline phase of the experiment did not induce adaptation, there were no significant differences in peak perpendicular error between baseline field trials and the very first field trials with the same arm in the initial learning phase for the abrupt field introduction groups (Fig. 4, *A* and *B*; all t_7_ < 1.9, all *P* > 0.98). As is the case for the channel force data, the behavior exhibited on the very first trials in the transfer phase of the experiment is critical to determine whether there was transfer of learning between limbs. In the case of kinematic errors induced by the force field, however, performance benefits could be due to feedforward compensation for the imposed dynamics, adaptive impedance tuning, or altered reflex gains. Errors exhibited by the transfer limb were not significantly different between baseline field trials and the very first field trial in the transfer phase for any abrupt condition (Fig. 4, *A* and *B*; all t_7_ < 1.7, all *P* = 1.0).

**Fig. 5.**
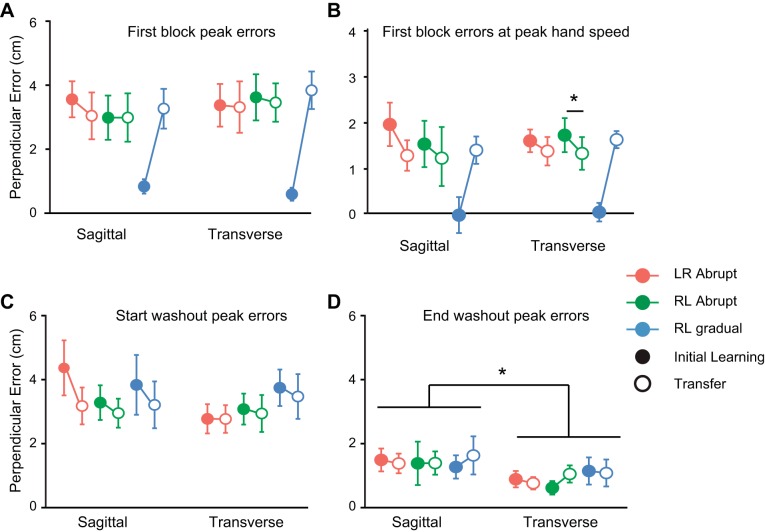
Summary plots for kinematic errors. *A*: averages and 95% confidence intervals for peak perpendicular errors in the first initial learning- and transfer-phase blocks for each condition. Lines join initial learning- and transfer-phase data for each condition. *B*: averages and 95% confidence intervals for perpendicular error at the time of peak hand speed in the first initial learning and transfer blocks for all 6 conditions. Averages and 95% confidence intervals for peak perpendicular errors in the first (*C*) and last (*D*) blocks of the washout phases of the experiment. *P < 0.05, statistically significant main effects between sagittal and transverse planes of movement or pairwise contrasts between initial learning and transfer phase performance within a group.

When maximum perpendicular errors, averaged over the first block of 12 force-field trials, were considered, there were also no differences between the initial learning and transfer phases of the experiment for any abrupt group (Fig. 5*A*; all t_7_ < 2.3, all *P* = 1.0). The data suggest that adaptation to the dynamic perturbation with one limb failed to confer kinematic performance benefits to the contralateral limb in any condition. These findings differ from previous reports of significant right-to-left (but not left-to-right) transfer of kinematic performance when the force field was introduced abruptly (Criscimagna-Hemminger et al. 2003; Joiner et al. 2013; Wang and Sainburg 2004). To investigate the reason for this discrepancy, we also analyzed the kinematic error at the time of peak velocity following Joiner and colleagues (2013). In this case, errors were reduced significantly for the left arm after prior right-arm exposure to a transverse force field (Fig. 5*B*; t_7_ = 4.5, *P* = 0.01), in agreement with previous studies (Criscimagna-Hemminger et al. 2003; Wang and Sainburg 2004). Transfer was not significant for the transverse left-to-right transfer condition (t_7_ < 1.8, *P* = 0.23) nor for either transfer direction for sagittal reaching (both t_7_ < 2.7, both *P* > 0.13), although it is possible that small but genuine differences in these conditions may have been obscured by a lack of statistical power. More critically, although the pattern of lateral asymmetry observed for the transverse plane replicates the kinematic error results of previous studies (Criscimagna-Hemminger et al. 2003; Wang and Sainburg 2004), the fact that there was no evidence of asymmetry for transfer measured with force channels in the current study demonstrates that transfer of force-field compensation is equally strong, irrespective of which arm is first exposed to the new dynamics.

#### Washout-phase results.

After both arms had extended exposure to the force field in the initial learning and transfer phases of the experiment, the arm first exposed performed 48 trials in the null field (with the robot motors turned off) to assess the “washout” of learning. The subjects then switched hands for a final time so that the transfer limb could also be tested in the null field. Perpendicular errors were significantly greater in the first washout phase involving the limb that first adapted to the field than for the transfer limb (Fig. 5*C*; F_1,42_ = 17.7, *P* < 0.001), and this effect was significantly stronger for the sagittal rather than the transverse conditions [plane of movement by arm (transfer arm vs. initial learning arm) interaction; F_1,42_ = 8.0, *P* = 0.007]. These results imply that a short bout of reaching in a null field with one limb reduces force-field compensation previously acquired by the contralateral limb. The perpendicular errors in the last washout blocks, pooled across phases and groups, were also larger for the sagittal- rather than the transverse-reaching conditions (Fig. 5*D*; F_1,42_ = 15.9, *P* < 0.001). This contrast remained significant if baseline errors were subtracted from the last-block washout errors (F_1,42_ = 5.0, *P* = 0.03), indicating that the effect cannot be explained simply by differences in baseline accuracy levels between tasks. Given that errors caused by random field trials during baseline were smaller for sagittal groups, this suggests that the internal representation of newly acquired dynamics or learned impedance control strategies are more persistent for sagittal than transverse plane reaching.

## DISCUSSION

The purpose of the current study was to determine the extent to which newly acquired dynamics can transfer to the opposite limb, given the context of recent data suggesting that such learning might be encoded according to multiple frames of reference (Berniker et al. 2014). Previous research on this topic exclusively involved force fields oriented in the transverse plane, where extrinsically defined fields have conflicting joint-based effects for the left and right limbs (Criscimagna-Hemminger et al. 2003; Joiner et al. 2013; Malfait and Ostry 2004; Wang and Sainburg 2004). Thus learning that is available to the opposite limb might not have enhanced performance in these studies because it was not represented in a compatible form. Our current study resolves this issue by aligning reference frames for both limbs via a field oriented in the sagittal plane, thereby excluding the possibility that substantial crosslimb availability was masked in previous studies by misalignments between intrinsic and extrinsic components of the learning. The finding that transfer is moderate at best in the absence of potential conflicts suggests that force-field learning is only weakly accessible to the opposite limb.

The current data also resolve the issue of whether transfer of force-field learning is asymmetrical. Previous papers, in which stronger right-to-left transfer was reported, did not use a force channel to assess transfer and therefore, could not exclude the effects of adaptive impedance changes and reflex tuning (Criscimagna-Hemminger et al. 2003; Wang and Sainburg 2004). We show that transfer of learned compensations for newly encountered dynamics is equally strong, irrespective of which arm is first exposed to the new dynamics.

### 

#### Are channel-trial forces really impervious to limb impedance changes?

Before considering the possible mechanisms and implications of our data further, we address the validity of our assumption that forces exerted orthogonal to the virtual channel from the start location to the target primarily reflect a learned compensation of the novel dynamics rather than adaptive limb impedance changes (Darainy et al. 2009; Milner and Franklin 2005; Toffin et al. 2003). We define adaptive impedance tuning as changes in muscle activation or posture that resist deviations from a straight path between the home and target locations but do not produce force orthogonal to the desired trajectory unless there is a trajectory deviation. According to this definition, impedance changes should have no effect on channel-trial behavior when tested under identical mechanical conditions. It remains possible, however, that learned muscle activation or limb configuration changes, which tune impedance to resist perturbation for a given movement, might generate non-negligible forces against the walls of a force channel in alternate mechanical conditions, such as reaching in a different direction or with the other limb. Nonetheless, channel-trial movements in our experiments were always to and from the central target, such that the required movements were very similar for the two limbs. If we assume that anatomical differences between the limbs are minor, then adaptive impedance changes should have had little impact on forces perpendicular to the direction of motion in either plane in our experiments.

We acknowledge that adaptive changes in muscle activation used to compensate the force field actively would also necessarily change the mechanical impedance of the limb and that application of similar muscle activations to the opposite limb would likely yield substantial perpendicular forces in channel trials. However, we consider such changes to reflect feedforward adaptation, because they compensate the force field directly by exerting forces orthogonal to the desired hand trajectory rather than resist deviation from the desired path, as would constitute “adaptive impedance tuning,” according to our definition. Thus we submit that adaptive changes in limb impedance likely contributed little to changes in channel-trial behavior.

#### Mechanism for enhanced sagittal plane transfer.

The finding that transfer to the opposite limb was greater after sagittal than transverse plane force-field adaptation suggests that transfer is impaired when the orientation of imposed dynamics misaligns between limbs in intrinsic coordinates. The results are therefore consistent with the hypothesis that learned dynamics are internally represented according to both the intrinsic and extrinsic coordinates of the sensorimotor context experienced during adaptation. According to this perspective, the extent of bilateral performance benefits observed in a given context will depend on both of the following: *1*) the extent to which the neural substrate of sensorimotor learning is accessible to the opposite limb and *2*) the degree to which the internal representation of a given perturbation is spatially compatible for the two limbs, according to multiple frames of reference.

In previous experiments involving transfer in viscous curl fields, it would appear that extrinsic representations of learning over-rode any intrinsic components, since the net transfer was always consistent with the extrinsic field orientation. However, it is important to note that it is not possible to determine the degree to which learning is available to the opposite limb under conditions of reference-frame misalignment. This is because there is an infinite set of possible weightings, according to which intrinsic and extrinsic components of learning could be combined to yield net transfer of any given magnitude. For example, with the assumption that learning is represented equally in extrinsic and intrinsic coordinates (so that each representation accounts for one-half of the learning) and a simple summation of effects, the net extrinsic transfer of ∼10%, reported by Joiner et al. (2013), could, in principle, reflect that 20% of the extrinsic component and 0% of the intrinsic component of learning were available to the opposite limb. Equally, a 10% net transfer could signify that 80% of the extrinsic component and 60% of the intrinsic component were available. In this case, we would have expected a large (∼70%) transfer between limbs in sagittal conditions when extrinsic and intrinsic reference frames were aligned. Thus part of the motivation for the current study was to test whether previous reports of weak transfer might have underestimated the availability of learning between limbs. Our finding that transfer is moderate at best in the absence of potential conflicts, however, reveals that force-field learning is only weakly accessible to the opposite limb.

If learning is represented according to both intrinsic and extrinsic coordinates, then another important consideration is how these different representations are combined to generate a single motor plan. Brayanov et al. (2012) presented data to suggest that intrinsic and extrinsic components of visuomotor rotation learning appear to be combined via multiplicative or gain-field integration rather than via summation of independent representations. Similarly, Yokoi et al. (2011) showed that motor primitives for the left and right limbs appear to be combined during bimanual control via gain-field encoding. Although these reports suggest that integration of intrinsic-extrinsic and left-right force-field learning might also be multiplicative, the current data provide no direct evidence on the issue.

However, although our results are clearly compatible with the interpretation that reduced transfer in the transverse plane is due to differences in the joint torques required to compensate for the imposed force field with each limb, several alternative explanations require consideration. In particular, because reaching was less accurate in the sagittal than the transverse plane at baseline, it is possible that differences in the reliability of limb end-point control in the sagittal and horizontal conditions affected the extent to which the source of errors induced by the force fields was attributed to the environment vs. the body. For example, Berniker and Kording (2008) proposed an error-source estimation model that could account both for dominant-nondominant limb directional asymmetries in transfer of dynamic adaptations reported previously (Criscimagna-Hemminger et al. 2003; Wang and Sainburg 2004) and apparent differences in the coordinates (i.e., extrinsic vs. intrinsic), in which learned dynamics generalize within and between limbs (Criscimagna-Hemminger et al. 2003; Shadmehr and Mussa-Ivaldi 1994; Wang and Sainburg 2004).

The rationale by which credit assignment factors can explain transfer asymmetry is that errors attributed to a particular limb should promote adaptation that is specific to that limb, whereas errors attributed to the world should result in adaptation that is more generally applicable (i.e., allowing transfer to the opposite side). It is difficult to predict how differences in the orientation of perturbing forces (horizontal vs. vertical) in the current study would have influenced internal estimates of the source of the consequent errors. On the one hand, if the internal representation of limb dynamics were less certain at baseline for sagittal plane reaching, as is suggested by the less-accurate and more-variable performance observed, then the errors experienced upon introduction of the sagittal force field should have been more likely to be misattributed to a change in the properties of the limb engaged in the task rather than to the external environment (Berniker and Kording 2008). Accordingly, this perspective on credit assignment would predict weaker transfer to the opposite limb for sagittal than transverse plane reaching. On the other hand, perturbations aligned with gravity might tend to be attributed to the external world or the tool being manipulated (in this case, the robot handle). Although the gravitational force is typically constant during a given movement in natural reaching contexts, gravity produces nonlinear joint torque dynamics during movement. To the extent that a velocity-dependent force field oriented in the sagittal plane resembles a perturbation of reaching, due to an unexpected change in gravitational load, it is possible that vertical force fields are more likely to be attributed to the external world than the body and thereby, contribute to the superior transfer between limbs that we observed for sagittal plane reaching.

Another consideration for the interpretation of our results relates to the fact that there were differences in the rate and completeness of adaptation between the sagittal and horizontal plane tasks for the limb first exposed to the force field. Less-complete learning of the task could obviously result in weaker transfer to the opposite limb, which could, in principle, explain the impaired transfer that we observed for transverse plane reaching. However, Joiner et al. (2013) reported that the completeness of adaptation did not affect the degree of transfer when they compared brief (15 trials, 56% compensation for the force field) with extended (160 trials, 77% compensation for the field) adaptation periods. Thus the slightly smaller differences in the final extent of adaptation that we observed between sagittal and transverse conditions (84% vs. 71%) would appear an unlikely explanation for the differences in transfer. Moreover, because we assessed transfer on the basis of probe channel trials before any opportunity for adaptation with the opposite limb, any general capacity for faster rates of compensation to sagittal rather than to transverse force fields cannot directly account for enhanced sagittal plane transfer. The lack of significant differences in transfer, observed between gradual and abrupt adaptation conditions, suggests that the rate at which the dynamics are learned does not seem to affect the extent of transfer independently from related factors, such as task difficulty. However, it remains possible that some factor responsible for the more-rapid adaptation to a sagittal plane force field also contributed to greater transfer. A potential candidate might be the degree of task-relevant variability expressed at baseline (Wu et al. 2014), since baseline kinematic performance was more variable for sagittal than transverse conditions. Wu et al. (2014) showed that the extent of task-relevant baseline variability predicts adaptation rate, and it is conceivable that whatever mechanisms underlie faster adaptation as a consequence of greater variability might also promote greater transfer to the opposite limb.

In summary, we favor the conclusion that transfer was impaired in the current study, because the orientation of imposed dynamics conflicted between limbs in intrinsic coordinates. However, because there were clear differences in baseline-reaching behavior and adaptation characteristics between sagittal and transverse plane contexts, we draw this conclusion with some caution.

#### Relation to previous findings.

The small but significant crosslimb transfer of newly acquired dynamics that we observed for transverse plane reaching (9%) is similar in magnitude to that reported by Joiner et al. (2013) (9–12%) and confirms that the internal representation of novel dynamics is partially accessible to the opposite limb. Here, we also showed that adaptation to a viscous force field with the nondominant limb generalizes significantly to the dominant limb, whereas Joiner et al. (2013) considered only dominant-to-nondominant limb transfer. This finding contrasts with the conclusions of previous studies that inferred transfer on the basis of kinematic errors induced by force fields or inertial loads [Criscimagna-Hemminger et al. (2003); Wang and Sainburg (2004); cf. Burgess et al. (2007)]. The discrepancy is likely due to the fact that multiple processes, including adaptive impedance tuning and reflex corrections, can contribute to a more direct end-point path to the target in the presence of a force field and so could have contributed to the previous findings of asymmetry. Indeed, we replicated the previously reported asymmetry in kinematic error reductions due to opposite-arm adaptation to novel dynamics. Joiner et al. (2013) argued that enhanced impedance control dominates the early kinematic benefits of opposite-limb adaptation to new dynamics, because early kinematic performance of the transfer arm was enhanced, irrespective of the orientation of the force field encountered in initial learning. Thus in combination with the results of Joiner et al. (2013), our current finding of symmetric transfer for channel-trial data, which reflect predominantly feedforward compensation for novel dynamics, suggests that asymmetries—in the degree to which dominant and nondominant limb force-field adaptation results in straighter movements when first exposed to the field with the opposite limb—are likely due to better transfer of impedance control strategies from the dominant to the nondominant limb.

It is notable that even when the force-field perturbation matched in both joint-based and extrinsic reference frames for the two limbs for sagittal reaching in the current study, transfer was still relatively week (i.e., ∼25%). This contrasts with our recent study on visuomotor rotation in an isometric aiming task (Carroll et al. 2014), where transfer between limbs was ∼70% when the perturbation was aligned in both intrinsic and extrinsic coordinates for the two limbs. Thus there seem to be fundamental differences in the interhemispheric generalizability of purely visuomotor recalibrations and learned adjustments to maps between kinematic states and joint torques or muscle forces. The weak force-field transfer observed indicates that a substantial component of the internal representation of novel dynamics is functionally inaccessible to the opposite limb. It remains possible, however, that part of the mechanism for the relative inability (i.e., despite the requirement for similar hand force and joint torques) for force-field learning, obtained with one limb to aid performance with its opposite, is that the neural substrate of force-field compensation is mediated via neural networks that are linked to the specific mechanical or neurophysiological properties of the limb used during initial learning (Raphael et al. 2010; Tsianos et al. 2014).

## GRANTS

Support for this work was provided by the Australian Research Council (Grant DP1093193), Trinity College, Wellcome Trust, Human Frontier Science Program, and Royal Society Noreen Murray Professorship in Neurobiology (to D. M. Wolpert).

## DISCLOSURES

The authors declare no potential conflicts of interest or competing financial interests.

## AUTHOR CONTRIBUTIONS

Author contributions: T.J.C., A.d.R., I.S.H., J.N.I., and D.M.W. conception and design of research; T.J.C. performed experiments; T.J.C. analyzed data; T.J.C. interpreted results of experiments; T.J.C. prepared figures; T.J.C. drafted manuscript; T.J.C., A.d.R., I.S.H., J.N.I., and D.M.W. edited and revised manuscript; T.J.C., A.d.R., I.S.H., J.N.I., and D.M.W. approved final version of manuscript.
